# The Role of High Fat Diets and Liver Peptidase Activity in the Development of Obesity and Insulin Resistance in Wistar Rats

**DOI:** 10.3390/nu12030636

**Published:** 2020-02-28

**Authors:** Germán Domínguez-Vías, Ana Belén Segarra, Manuel Ramírez-Sánchez, Isabel Prieto

**Affiliations:** 1 Unit of Physiology, Department of Health Sciences, University of Jaén, Las Lagunillas, 23071 Jaén, Spain; germandv@go.ugr.es (G.D.-V.); asegarra@ujaen.es (A.B.S.); msanchez@ujaen.es (M.R.-S.); 2 Department of Physiology, Faculty of Health Sciences, Ceuta, University of Granada, 18071 Granada, Spain

**Keywords:** obesity, high-fat diet, olive oil, aminopeptidase activity, renin–angiotensin-system

## Abstract

High-fat diets (HFD) have been widely associated with an increased risk of metabolic disorders and overweight. However, a high intake of sources that are rich in monounsaturated fatty acids has been suggested as a dietary agent that is able to positively influence energy metabolism and vascular function. The main objective of this study was to analyze the role of dietary fats on hepatic peptidases activities and metabolic disorders. Three diets: standard (S), HFD supplemented with virgin olive oil (VOO), and HFD supplemented with butter plus cholesterol (Bch), were administered over six months to male Wistar rats. Plasma and liver samples were collected for clinical biochemistry and aminopeptidase activities (AP) analysis. The expression of inducible nitric oxide synthase (iNOS) was also determined by Western blot in liver samples. The diet supplement with VOO did not induce obesity, in contrast to the Bch group. Though the VOO diet increased the time that was needed to return to the basal levels of plasma glucose, the fasting insulin/glucose ratio and HOMA2-%B index (a homeostasis model index of insulin secretion and valuation of β-cell usefulness (% β-cell secretion)) were improved. An increase of hepatic membrane-bound dipeptidyl-peptidase 4 (DPP4) activity was found only in VOO rats, even if no differences in fasting plasma glucagon-like peptide 1 (GLP-1) were obtained. Both HFDs induced changes in hepatic pyroglutamyl-AP in the soluble fraction, but only the Bch diet increased the soluble tyrosyl-AP. Angiotensinase activities that are implicated in the metabolism of angiotensin II (AngII) to AngIV increased in the VOO diet, which was in agreement with the higher activity of insulin-regulated-AP (IRAP) in this group. Otherwise, the diet that was enriched with butter increased soluble gamma-glutamyl transferase (GGT) and Leucyl-AP, iNOS expression in the liver, and plasma NO. In summary, VOO increased the hepatic activity of AP that were related to glucose metabolism (DPP4, angiotensinases, and IRAP). However, the Bch diet increased activities that are implicated in the control of food intake (Tyrosine-AP), the index of hepatic damage (Leucine-AP and GGT), and the expression of hepatic iNOS and plasma NO. Taken together, these results support that the source of fat in the diet affects several peptidases activities in the liver, which could be related to alterations in feeding behavior and glucose metabolism.

## 1. Introduction

Obesity is considered a serious chronic disease with adverse consequences due to the excessive accumulation of adipose tissue. Usually, obesity is caused by an imbalance between energy intake and energy expenditure [1]. The prevalence of obesity in the population has increased alarmingly in the past few decades, and it typically it is a part of a cluster of metabolic syndrome conditions that increase the chance of developing several related disorders, such as type 2 diabetes, dyslipidemia, abnormal cholesterol levels, hypertension, stroke, heart disease, and cancer [2,3]. Several studies have documented a positive relationship between a high-fat diet (HFD) intake and obesity [4]. The efficacy of the current strategies of treatment for diseases that are caused by obesity is still not entirely satisfactory, and new approaches must be considered. Several studies have indicated relationships between the saturation of dietary fat and the concentrations of total plasma cholesterol and higher blood pressure [5], hepatic steatosis [6], adipose chronic inflammation [6], ectopic lipid deposition in the liver and brown fat [6], overweight [6,7], hyperinsulinemia [7], and hepatic insulin resistance [8]. However, high fat diets that are rich in monounsaturated fatty acids exert a protective role [9,10,11,12,13,14,15].

High monounsaturated fatty acids diets also have demonstrated an effect on the secretion of the insulinotropic peptide GLP-1 (glucagon-like peptide 1) [16,17]. GLP-1 is an incretin hormone with antidiabetic action that is capable of stimulating insulin secretion [18,19], increasing beta cell neogenesis [20], inhibiting beta cell apoptosis [20], inhibiting glucagon secretion [21], delaying gastric emptying, and inducing satiety [20,21]. However, the physiological inhibitory control of GLP-1 and its accelerated inactivation by plasma dipeptidyl-peptidase 4 (DPP4) hyperactivity [22] suggests that the impaired secretion and/or activity of GLP-1 may be involved in the pathogenesis of obesity [23].

The renin–angiotensin system (RAS) is an important regulator of blood pressure, electrolytes, and water balance. Until recently, angiotensin II (Ang II) was considered the main peptide that is involved in these homeostatic mechanisms. However, some of its metabolic derivatives such as angiotensin III (Ang III), angiotensin IV (Ang IV), and angiotensin 2-10 (Ang 2-10) have demonstrated important biological functions [24] (Figure 3A). The main peptides of the RAS (AngII and AngIII) bind to both AT1- and AT2-receptors with similar affinity [25,26]. In contrast, the affinity of Ang IV for the well-characterized AT1- and AT2-receptors is low [25], but it shows a high affinity to the AT4-receptor/insulin-regulated aminopeptidase (IRAP) [27]. Ang IV, which binds to IRAP (Figure 3A), appears to play a role in regulating local blood flow [28]. Local RASs could be altered by different degrees of saturation in dietary fat. Angiotensin peptides are metabolized by several angiotensinases, and we have previously demonstrated that those activities are affected by dietary fatty acids [5,29,30].

The local RAS in the liver plays an important role in the paracrine and autocrine regulation of hepatocyte metabolism [31], and all the components of this system have been described in hepatic cells [32,33,34]. Under pathological conditions, the role of the liver’s RAS appears to be more important. Local angiotensin peptides increase cell proliferation, apoptosis, and the production of oxygen reactive species [35]. The Western diet induces liver steatosis and is also associated with the alteration of the local RAS in hepatocytes [36], and both renal and hepatic local RASs seem to be implicated in the development of chronic liver disease [37].

Lipids are recognized to be associated with the intracellular generation of reactive oxygen species (ROS) [38] as support for oxidative damage. The HFD, and subsequent dyslipidemia, are the major triggers for oxidative stress, and the increase of gamma-glutamyl transferase (GGT) like hepatic function biomarker [39]. Nitric oxide (NO) exerts a protective effect on liver diseases and hepatotoxicity that is associated with HFD [40,41]. The hepatic inducible isoform of the NO synthase (iNOS) functions as an adaptive response to minimize inflammatory injury [40,41].

The thyrotropin-released hormone (TRH)-degrading pyroglutamate aminopeptidase (pGluAP) activities convert TRH into cyclo His-Pro (cHP) [42], a potential hypoglycemic dipeptide that restores glucose metabolism, the blood insulin level, the lipid profile, and impaired β-cells in the pancreas [43]. Previous results suggested that the type of fat that is used in the diet may influence local pGluAP activity and modify its biological functions [44]. Other studies have implicated opioid transmission in the hedonic and metabolic control of feeding and diet-induced obesity [45]. Enkephalin (ENK) concentration changes in liver tissue and plasma during liver disorders [46,47], after a high-fat meal with an increased propensity to overeat [48], and with a higher systolic blood pressure [49]. ENK is hydrolyzed by specific enzymes, such as the ENK-degrading tyrosyl aminopeptidase (TyrAP) [50]. Then, hepatic TyrAP activity could be an index to evaluate the potential value of ENK degradation as a biological marker of the hedonic control and progression of obesity disorders.

In order to analyze the effects of HFD on liver peptide metabolism, we administered diets with different amounts and fat sources to rat groups in this study. We demonstrate that monounsaturated HFD is not related to metabolic manifestations because overweight, dyslipidemia, and high cholesterol levels were present in the animals that were fed with a saturated HFD. However, monounsaturated HFD mainly affects parameters that are related to glucose metabolism, probably in agreement with a greater use of fatty acids for energy. At the same time, the analyzed diets had a differential effect on several aminopeptidase activities with important roles in the development of metabolic syndromes.

## 2. Materials and Methods

### 2.1. Animals and Treatments

Adult male Wistar rats were purchased from Harlan Interfauna Ibérica S.A. (Barcelona, Spain). The rats had free access to experimental diets and water during 24 weeks, and they were maintained at a controlled temperature (20–25 °C) and humidity (50 ± 5%) environment in a 12 hours light/dark cycle. At the beginning of the study, the mean body weight and age were 495 g and six months, respectively. Experimental procedures for animal use and care were in accordance with European Communities Council Directive 2010/63/UE and Spanish regulation RD 53/2013, and the study was approved by the Institutional Animal Care and Use Committee of the University of Jaén. Rats were randomly assigned into three groups (*n* = 5 each): In the standard diet (S) group, rats were fed with a commercial chow for experimental animals. In HFDs, one group of rats was fed with a diet supplemented with 20% of virgin olive oil (VOO), and the second group of rats was fed with a diet supplemented with 20% butter plus cholesterol (0.1%) (Bch) in order to reach the average cholesterol content of the Western diet. The HFD diets were isocaloric. The food composition and nutritive value of different diets are shown in Supplemental Table S1. The food intake for each group was measured, and the animals were weighed once a week. At the end of the experimental period, a glucose tolerance test was performed. Animals were perfused with a saline solution through the left cardiac ventricle under Equithensin anesthesia (2 mL/kg Body Weight), and a sample of blood was collected for GLP-1, insulin, NO and other biochemical parameters. The blood samples were centrifuged for 10 min at 2000 *g* to obtain the plasma. Liver tissue samples were collected for angiotensinase activities, ENK- and TRH-degrading activities, hepatic damage markers, and immunoblot for hepatic iNOS.

### 2.2. Glucose Tolerance Test

A glucose tolerance test (GTT) was performed after overnight fasting. For the GTT, rats were i.p. injected (8 mL/kg BW) with a single dose of glucose that was dissolved in saline (2.0 g/kg BW). Blood glucose was measured by using a glucometer (Roche Accu-Check Inform.). A baseline (fasting) blood glucose measurement was taken before glucose administration, and further measurements were made at regular intervals thereafter (20, 40, 60 and 90 min).

### 2.3. Determination of Blood Parameters

Plasma insulin levels were determined by using an ELISA kit (#10-1113-01) that was purchased from Mercodia Developing Diagnostics (Winston Salem, NC, USA). Plasma GLP-1 concentrations were determined by using an ELISA kit (#107444-51-9) that was purchased from Cayman (Ann Arbor, MI, USA).

Plasma levels of NO were analyzed by the Griess method as a summation of NO2- and NO3- with an assay kit (Total Nitric Oxide and Nitrate/Nitrite Parameter#KGE001) that was purchased from R&D Systems (Minneapolis, MN, USA).

### 2.4. Determination of Insulin Resistance

A homeostasis model of insulin resistance (HOMA2-IR) was calculated by using the HOMA Calculator v.2.2.3 software [51]. HOMA-2 is an update and actualization of the HOMA equation, where a lineal regression is established between glucose and insulin by adjusting real physiology. It is possible, with this program, to calculate the HOMA2-%B, an index of insulin secretion and a valuation of β-cell usefulness (% β-cell secretion), and to know HOMA2-%S, an index for estimating sensitivity to insulin (% insulin sensitivity).

### 2.5. Sample Preparation for Aminopeptidase Activities Assay

Plasma samples were used for the peptidase activities assay. Samples from the liver were quickly removed and frozen in dry ice. To obtain a soluble fraction, tissue samples were homogenized in ten volumes of a 10 mM HCl-Tris buffer (pH 7.4) and ultracentrifuged (100,000 *g* for 30 min at 4 °C). The resulting supernatants were used to measure soluble (sol) enzymatic activity and protein content, assayed in triplicate. To solubilize membrane proteins, pellets were re-homogenized in an HCl-Tris buffer (pH 7.4) plus Triton X-100 (1%). After centrifugation (100,000 *g* for 30 min at 4 °C), supernatants were used to measure membrane-bound (mb) activity and proteins in triplicate. To ensure the complete recovery of activity, detergent (Triton X-100) was removed from the medium by adding the adsorbent polymeric SM-2 Biobeads (100 mg/mL), which were purchased form Bio-Rad (Richmond, VA, USA), to the samples and the shaking for 2 h at 4 °C [52].

### 2.6. Peptidase Activities Assay

The DPP4 family, GGT, LeuAP, pGluAP, TyrAP and angiotensinase (AspAP, GluAP, AlaAP, and IRAP) activities were determined in a fluorometric assay while using arylamidase as substrate according to the method of Ramírez et al. [53]. The substrate and substrate solution for each activity are listed in Supplemental Table S2. Briefly, 10 µL of each supernatant were incubated for 30 min at 37 °C with 100 µL of the substrate solution [54]. Later, enzymatic reactions were ceased after adding 100 µL of a 0.1 M acetate buffer (pH 4.2). The β-naphthylamine (β-NA) that was released as a product of proteolytic activity was fluorometrically assessed at 412 nm emission and at a 345 nm excitation wavelength. Specific peptidases activities were expressed as pmol of the β-NA that was hydrolyzed per minute and per milligram of protein. Fluorogenic assays were linear with respect to time of hydrolysis and protein concentration. The protein concentration was determined according to Bradford method [55] with BSA (Bovine Serum Albumin) as standard. All chemical products were supplied by Sigma (St. Louis, MO, USA).

### 2.7. Protein Extraction and Western Blot Analysis

For the Western blotting, total protein was isolated from the liver samples. The hepatic tissues were homogenized in a cooled protein extraction buffer (Tris-HCl, 0.1 M, pH 7.5; aprotinin, 0.1 mg/mL; sodium pyrophosphate, 0.1 M; sodium fluoride, 0.1 M; EDTA (Ethylene Diamine Tetraacetic Acid), 0.01 M; sodium orthovanadate, 0.01 M; and PMSF (Phenyl Methyl Sulfonyl Fluoride), 0.002 M), mixed with 10% Triton X-100 and centrifuged (12,000 *g* for 30 min at 4 °C). The supernatants were collected, and then the protein contents were analyzed with a micro Lowry reaction (DCTM Protein Assay #500-0116) purchased from Bio-Rad Laboratories (Hercules, CA, USA). A Western blot analysis was performed by using iNOS antibodies (BD Biosciences, CA, USA). The same amount of extracted protein from each sample (30~40 µg) was loaded for the sodium dodecyl sulphate-polyacrylamide gel electrophoresis (SDS-PAGE) immunoblot analysis. The separated proteins were electrophoretically transferred into a nitrocellulose membrane (Amersham), which was then blocked with 5% BSA and 0.1% Tween for 60 min at room temperature. The membranes were incubated with the primary antibody overnight at 4 °C. The regions containing proteins were visualized by using ECL (Amersham ECL prime Western blotting detection reagent # RPN2232) purchased from GE Healthcare Life Sciences (Buckinghamshire, UK). Each band was normalized by the corresponding value of α1-tubulin as an internal control. A densitometric analysis was performed by Image J 1.36b (NIH) Software. The primary antibodies against the proteins are listed in Table S3.

### 2.8. Statistical Analysis

A statistical analysis was performed by one-way ANOVA followed by Tukey’s post-hoc test for multiple comparisons. A comparison of parameters between two groups was performed with an unpaired Student’s t-test. The relationship between variables were assessed by Pearson’s correlation coefficient. The presence of significant difference was estimate with the Sigmaplot v11.0 software (Systat Software, Inc., San Jose, CA, USA), and *p* values below 0.05 (*p* < 0.05) were considered statistically significant. All the data are presented as mean ± standard error of the mean (SEM).

## 3. Results

### 3.1. Food and Energy Intakes

**Figure 1:**
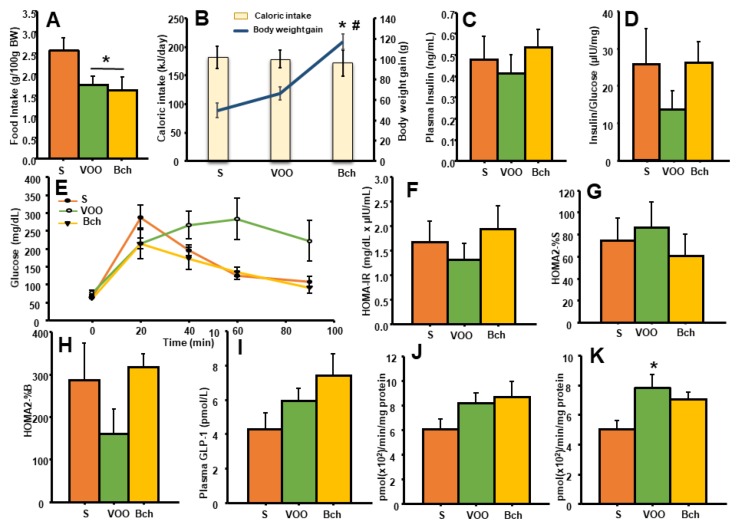
Means values ± standard errors of daily food intake, expressed as g/100 g Body Weight (**A**); the intake of energy, expressed as kJ/day and total body weight gain, expressed as g (**B**); values of plasma fasting insulin, expressed as ng/mL (**C**); ratio of plasma fasting insulin/glucose, expressed as µIU/mg (**D**); values of glucose during test tolerance (GTT), expressed as mg/dL (**E**); homeostasis model of insulin resistance (HOMA-IR), expressed as mg/dL x µIU/mL (**F**); an index for estimating sensitivity to insulin (% insulin sensitivity) (HOMA2-%S) (**G**); an index of insulin secretion and a valuation of β-cell usefulness (% β-cell secretion) (HOMA2%B) (**H**); plasma glucagon-like peptide 1 (GLP-1), expressed as pmol/L (**I**); and dipeptidyl-peptidase 4 (DPP4) activity in the liver-soluble fraction (**J**) and membrane-bound fraction (**K**), expressed as pmol/min/mg prot. S: standard diet, VOO: virgin olive oil diet, Bch: butter plus cholesterol diet. * indicates significant differences between VOO or Bch vs. S, * *p* < 0.05. # indicates significant differences between VOO and Bch, # *p* < 0.05.

A significant difference was observed between the S and HFDs groups for daily food intake (g/100g BW), with the lowest values found for the Bch-fed rats (Figure 1A: Bch, 1.6 ± 0.3 g; VOO, 1.8 ± 0.2 g; S, 2.6 ± 0.3 g; *p* < 0.05). However, not significant differences for energy intake (kJ/day) were observed between the groups (Figure 1B: S, 181.8 ± 19.9; VOO, 178.2 ± 15.4; Bch, 171.4 ± 22.8), while the total weight gained was significantly higher in the Bch group (Bch, 151.9 ± 10.0 g; VOO, 101.6 ± 6.2 g; S, 73.6 ± 10.3 g; *p* < 0.05).

### 3.2. Glucose Tolerance Test (GTT) and Glycemic Control

No significant differences were found between groups in fasting plasma glucose (Figure 1E: S, 63.8 ± 7.6 mg/dL; VOO, 83 ± 13 mg/dL; Bch, 63 ± 2.5 mg/dL) and insulin (Figure 1C: S, 0.5 ± 0.1 ng/mL; VOO, 0.4 ± 0.1 ng/mL; Bch, 0.5 ± 0.1 ng/mL;). The effects of different diets on glucose tolerance were established at the end of the experimental period by using GGT (Figure 1D).

The highest levels of plasma glucose were achieved after 20 min of i.p. injection in the S and Bch groups (Figure 1E: S, 286.7 ± 34.9 mg/dL; VOO, 214.5 ± 43.4 mg/dL; Bch, 214.8 ± 15.7 mg/dL). Nevertheless, the VOO animals achieved the highest plasma glucose at 60 min after i.p. injection (S, 124.5 ± 11.7 mg/dL; VOO, 282.8 ± 58.5 mg/dL; Bch, 135 ± 12.3 mg/dL), and it remained elevated at 90 min.

Regarding the homeostasis model of insulin resistance, no significant differences were found between the diets in the fasting ration of insulin/glucose (Figure 1D: S, 25.8 ± 9.5 µIU/mg; VOO, 13.7 ± 5.0 µIU/mg; Bch, 26.3 ± 5.5 µIU/mg), HOMA-IR (Figure 1F: S, 1.7 ± 0.4; VOO, 1.3 ± 0.3; Bch, 1.9 ± 0.5), HOMA2-%S (Figure 1G: S, 74.8 ± 19.8; VOO, 86.6 ± 22.6; Bch, 60.7 ± 19.1) or HOMA2-%B (Figure 1H: S, 286.5 ±87.7; VOO, 159.6 ± 58.9; Bch, 317.2 ± 31.6).

### 3.3. Glucagon-Like-Peptide-1 and Dipeptidyl-Peptidase- 4 Activity

Though significant differences were not observed in circulating GLP-1 levels between the groups (Figure 1I: S, 4.3 ± 1.0 pmol/L; VOO, 6.0 ± 0.7 pmol/L; Bch, 7.4 ± 1.3 pmol/L), the hepatic DPP4 family activity (Figure 1J–K) was significantly higher in the high fat diets than in the control group. Namely, the hepatic membrane-bound fraction showed the significantly highest values in the VOO-fed rats compared with the S and Bch groups. (Figure 1K: S, 5.0 ± 0.5 pmol (×10^2^)/min/mg protein; VOO, 7.8 ± 0.8 pmol (×10^2^)/min/mg protein; Bch, 7.0 ± 0.4 pmol (×10^2^)/min/mg protein) (Figure 1L: S, 0.8 ± 0.1 pmol (×10^2^)/min/mg protein; VOO, 1.4 ± 0.2 pmol (×10^2^)/min/mg protein).

### 3.4. Hepatic TRH-Degrading Pyroglutamate Aminopeptidase Activity and Tyrosine Aminopeptidase Activity

**Figure 2:**
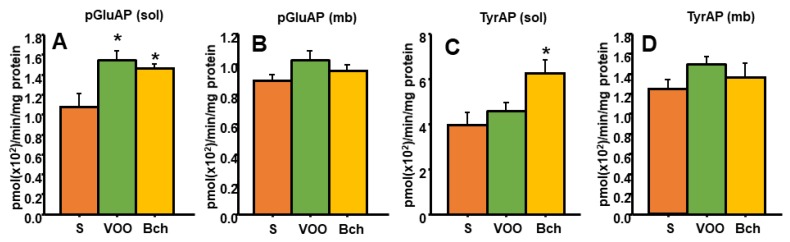
Means values ± standard errors of pyroglutamyl aminopeptidase activity (pGluAP) in liver-soluble (**A**) and membrane-bound (**B**) fractions, and tyrosine aminopeptidase activity (TyrAP) in liver-soluble (**C**) and membrane-bound (**D**) fractions, expressed as pmol/min/mg prot. S: standard diet, VOO: virgin olive oil diet, Bch: butter plus cholesterol diet. * indicates significant differences between VOO or Bch vs. S. * *p* <0.05.

Previous studies have demonstrated that the effects of thyroid status on hepatic pGluAP and the release of endogenous peptides as cyclo His-Pro (cHP) are determinant in insulin sensitivity and body weight control [56,57]. In our results, HFDs significantly increased the soluble pGluAP activity in the liver (Figure 2A: S, 1.0 ± 0.1 pmol (×10^2^)/min/mg protein; VOO, 1.5 ± 0.0 pmol (×10^2^)/min/mg protein; Bch, 1.4 ± 0.0 pmol (×10^2^)/min/mg protein). However, no differences were found in membrane-bound activity (Figure 2B).

On the other hand, enkephalins have been widely implicated in feeding behavior, and these endogenous opioid peptides are hydrolyzed by the tyrosine aminopeptidase activity (TyrAP). Our results showed higher levels of soluble tyrosyl aminopeptidase activity in the liver of rats that were fed with the Bch diet (Figure 2: S, 3.9 ± 0.5 pmol (×10^2^)/min/mg protein; VOO, 4.5 ± 0.3 pmol (×10^2^)/min/mg protein; Bch, 6.2 ± 0.5 pmol (×10^2^)/min/mg protein), but no significant differences were found in the membrane-bound fraction.

### 3.5. Hepatic Angiotensinases Activities

**Figure 3:**
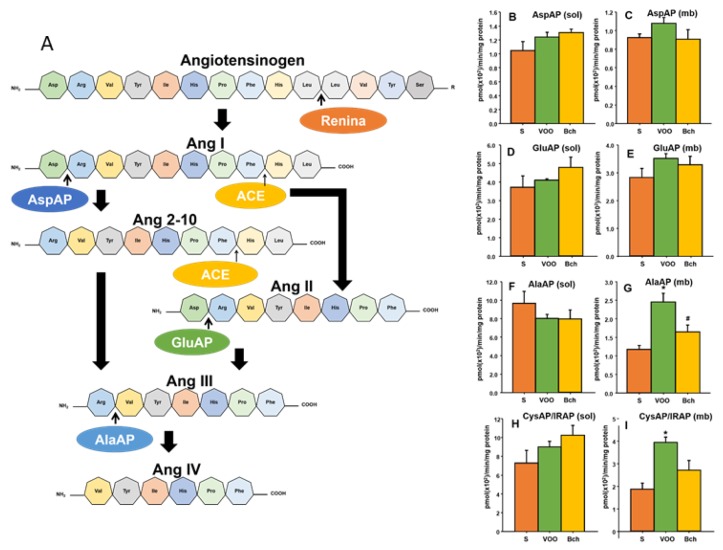
Partial representation of the renin–angiotensin system that shows the metabolic steps in which angiotensinase activities are involved (**A**); means values ± standard errors of aspartyl aminopeptidase activity (AspAP) in liver-soluble (**B**) and membrane-bound (**C**) fractions; glutamyl aminopeptidase activity (GluAP) in liver-soluble (**D**) and membrane-bound (**E**) fractions; alanyl aminopeptidase activity (AlaAP) in liver-soluble (**F**) and membrane-bound (**G**) fractions; and cystinyl aminopeptidase activity (CysAP) in liver-soluble (**H**) and membrane-bound (**I**) fractions expressed as pmol/min/mg prot. S: standard diet, VOO: virgin olive oil diet, Bch: butter plus cholesterol diet. * indicates significant differences between VOO or Bch vs. S, * *p* < 0.05. # indicates significant differences between VOO and Bch, # *p* < 0.05. ACE: angiotensin converting enzyme; IRAP: insulin regulated aminopeptidase.

Figure 3 shows the metabolism of angiotensin peptides in the local renin–angiotensin system, as well as the role of several aminopeptidases activities in soluble and membrane-bound fractions (AspAP, GluAP, AlaAP and CysAP/IRAP). Significant differences were not observed in soluble or membrane-bound fractions for aspartyl and glutamyl aminopeptidase activities between the diets (Figure 3B–E). However, the VOO diet significantly increased the activity of alanyl aminopeptidase activity (Figure 3G: S, 1.2 ± 0.1 pmol (×10^2^)/min/mg protein; VOO, 2.4 ± 0.2 pmol (×10^2^)/min/mg protein; Bch, 1.6 ± 0.2 pmol (×10^2^)/min/mg protein) and cystinyl aminopeptidase activity (Figure 3I: S, 1.9 ± 0.3 pmol (×10^2^)/min/mg protein; VOO: 3.9 ± 0.2 pmol (×10^2^)/min/mg protein; Bch: 2.7 ± 0.4 pmol (×10^2^)/min/mg protein) in the liver membrane-bound fraction.

### 3.6. Leucine Aminopeptidase and Gamma Glutamyl Transferase

**Figure 4:**
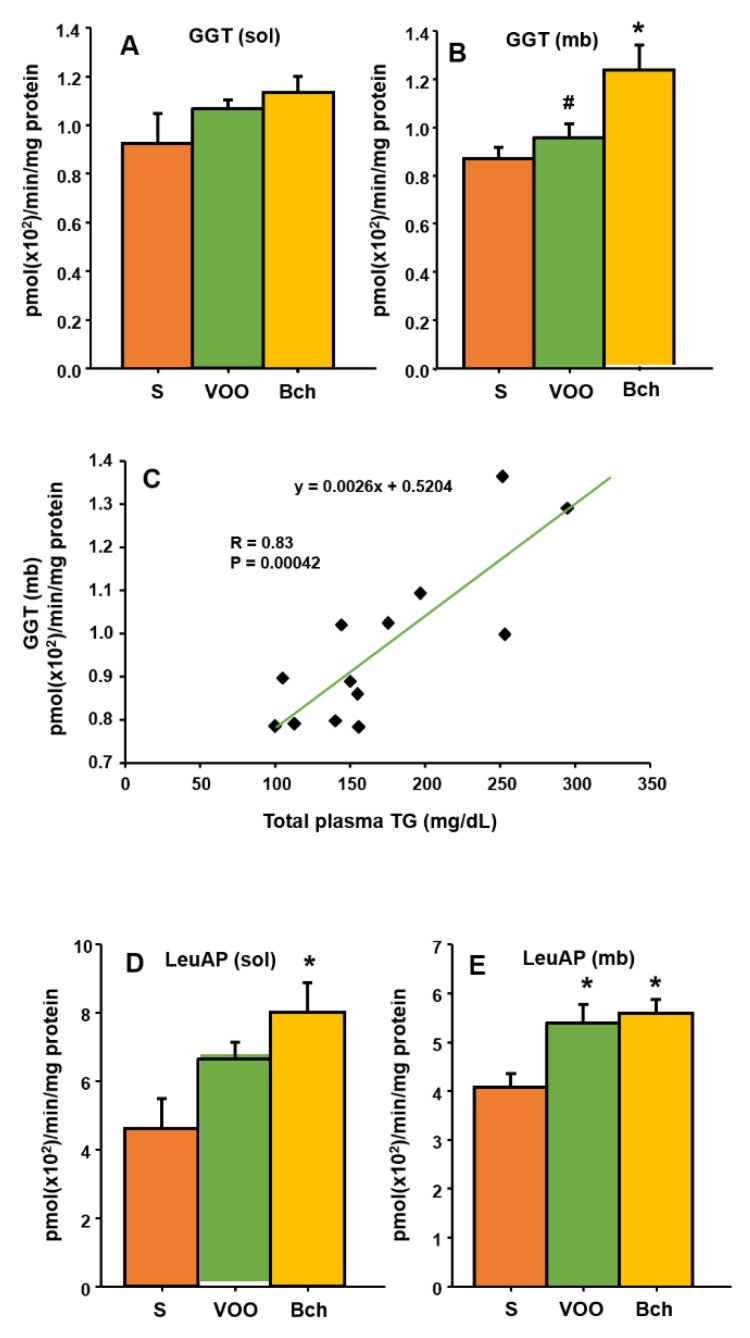
Means values ± standard errors of gamma glutamyl transferase activity (GGT) in liver-soluble (**A**) and membrane-bound (**B**) fractions, expressed as pmol/min/mg prot., linear regressions established between GGT and plasma triglycerides (TG) (**C**); and leucyl aminopeptidase activity (LeuAP) in liver-soluble (**D**) and membrane-bound (**E**) fractions, expressed as pmol/min/mg prot. S: standard diet, VOO: virgin olive oil diet, Bch: butter plus cholesterol diet. * indicates significant differences between VOO or Bch vs. S, * *p* < 0.05. # indicates significant differences between VOO and Bch, # *p* < 0.05.

Several indicators of hepatic function, such as GGT and leucine aminopeptidase (LeuAP), were evaluated (Figure 4A–E). The butter plus cholesterol diet significantly increased hepatic membrane-bound GGT activity compared with the S and VOO diets (Figure 4B: S, 0.8 ± 0.0 pmol (×10^2^)/min/mg protein; VOO, 09 ± 0.0 pmol (×10^2^)/min/mg protein; Bch, 1.2 ± 0.1 pmol (×10^2^)/min/mg protein). Furthermore, a significant relationship was established between GGT membrane-bound activity and the plasma lipid profile (total triglyceride) (Figure 4C).

Soluble LeuAP activity also increased in the Bch group (Figure 4D: S, 4.6 ± 0.8 pmol (×10^2^)/min/mg protein; VOO, 6.6 ± 0.4 pmol (×10^2^)/min/mg protein; Bch, 7.9 ± 0.8 pmol (×10^2^)/min/mg protein), whereas membrane-bound LeuAP activity was higher in the two HFDs (Figure 4E: 4.0 ± 0.2 pmol (×10^2^)/min/mg protein; VOO, 5.3 ± 0.3 pmol (×10^2^)/min/mg protein; Bch, 5.5 ± 0.2 pmol (×10^2^)/min/mg protein).

### 3.7. Plasma Nitric Oxide (NO) and Hepatic Inducible NO Synthase (iNOS)

**Figure 5:**
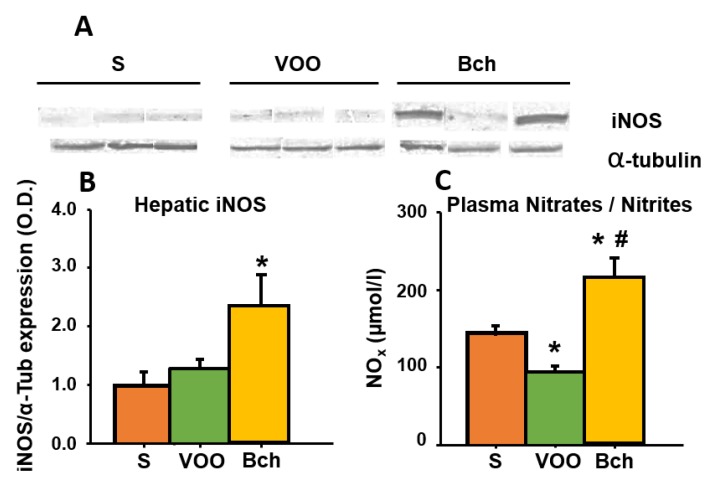
Hepatic inducible nitric oxide synthase (iNOS) measured by using by immunoblots (**A**), mean ±standard error of expression levels of iNOS that were quantified and normalized by the expression of α1-tubulin (**B**), and plasma NOx (NO2- and NO3-) concentration expressed as µmol/L (**C**). S: standard diet, VOO: virgin olive oil diet, Bch: butter plus cholesterol diet. * indicates significant differences between VOO or Bch vs. S, * *p* < 0.05. # indicates significant differences between VOO and Bch, # *p* < 0.05.

In order to investigate the effects of HFDs on oxidative stress, the expression of hepatic iNOS was determined by using a Western blot assay (Figure 5A,B), and the plasma NO concentrations (NOx) were measured by the conversion of nitrate (NO3-) in to nitrite (NO2-) by nitrate reductase (Figure 5C). The Bch-fed rats exhibited a significant increase in iNOS expression (Figure 5B: S, 1.0 ± 0.2; VOO, 1.3 ± 0.1; Bch: 2.3 ± 0.5). Moreover, the concentration NOx was significantly higher in the Bch group compared with the S and VOO groups. Interestingly, the VOO group showed lower NOx levels than the S group (Figure 5C: S, 1.4 ± 0.1 µmol/L; VOO, 0.9 ± 0.0 µmol/L; Bch, 2.1 ± 0.2 µmol/L).

## 4. Discussion

High fat diets have demonstrated the ability to be a good model of obesity and metabolic syndrome in laboratory animals models [6,7,8,56,58,59]. However, the efficiency in the use of energy also influences the development of obesity in rodents. A study by Liu [7] demonstrated that obesity development was influenced by gender and the efficiency in the use of energy. In the current study, the animals were fed with two isoenergetic HFDs but with different degree of saturation in their fatty acids (mainly monounsaturated in the VOO group and saturated in the Bch group) and minor components (polyphenols in the VOO group and cholesterol in the Bch group). While the Bch animals achieved a significantly higher body weight than the control group, the VOO rats did not show significant differences with the control group (data not shown). Nevertheless, the greater gain of body weight in the Bch rats were not associated with a higher food intake. Indeed, daily food intakes (g/100g BW) were lower in the two HFDs at the end of the experimental period.

Regardless of the energetic-density of the diet, animals are usually able to adapt their food intake and get the same amount of energy [60]. The differences in body weight gain between the HFDs may be explained by the fact that saturated fatty acids show higher levels of energetic efficiency and are able to decrease the mitochondrial oxidative capacity in the liver and the skeletal muscle [61]. However, the inclusion of VOO in the diet seems to have beneficial effects on body weight control [62,63], increase diet-induced thermogenesis [64], regulate UCP1 (uncoupling protein 1) expression in the adipose tissue [65], and enhance thermogenesis by increasing the UCP1 content in brown adipose tissue and increasing noradrenaline and adrenaline secretions [66]. These results suggest that the unsaturated dietary fatty acids and the quantity of dietary fat may have a significant effect on the regulation of thermogenic conditions. At the same time, these effects could be related to the polyphenols present in VOO [13,67].

The differences in final body weight do not seem to be associated with altered values of fasting plasma glucose and insulin in the three groups of animals. Nevertheless, our results showed differences in the intraperitoneal glucose tolerance test (GTT). During the GTT, the higher levels of plasma glucose were reached at 20 min after i.p. injection in the S and Bch groups. However, in VOO animals, the maximum values were delayed until 90 min after i.p. injection, and they remained high at 120 min. These results contradict with previous works [7,8,60], but the different index of insulin resistant that was calculated in our animals (HOMA-IR, HOMA-%S and HOMA-%B) were similar in the three diets. Still, mean values of fasting insulin/glucose and the HOMA2-%b were lower in the VOO group.

In the fed (postprandial) state, GLP-1 peptide has the ability to stimulate insulin secretion and the synthesis of glycogen in the liver, thus reducing postprandial hyperglycemia. On the other hand, the physiological inhibitory control of GLP-1 on gastric emptying and its contribution to the regulation of food intake and satiety suggests that impaired secretion and/or activity of GLP-1 may be involved in the pathogenesis of obesity. A previous study indicated that, in morbid obesity, the faster inactivation of circulating GLP-1 could result in lower plasma levels of this peptide and could contribute to eating behavior abnormalities [23]. Plasma DPP4 activity is the main factor that is implicated in the metabolism of GLP-1, as well as its main regulator [23,68]. In the present study, an increase of the hepatic DPP4 family activity was only found in the VOO-fed rats, even if no differences in fasting plasma GLP-1 levels were obtained between the three diets, probably because of the rapid inactivation of this peptide. Nevertheless, the high DPP4 activity in the VOO animals could have been related to high postprandial GLP-1 levels, which in agreement with other previous works [17,69,70]. During the GTT, the stimulation of the intestinal L cell did not take place, explaining the higher glucose values in the VOO group.

In addition to GLP-1, another peptide with important antidiabetic role able to improve insulin sensitivity and body weight control is cyclo-histidine-proline (cHP) [43,71]. Cyclo-histidine-proline can be found in hepatic cells [42,57], and it is a metabolic product of TRH by the activity of pyroglutamate aminopeptidase (pGluAP) [57].

The role of pGluAP and cHP in the obesity is not yet clear, but cHP plus zinc is effective in decreasing blood glucose concentrations in genetically obese (*ob*/*ob*), type 2 diabetic mice [72]. Moreover, it has been reported that HFDs raise TRH expression [73] and alter pGluAP activity, effects that seem to depend on the fat source [44]. Our results indicated that HFDs induced changes in hepatic TRH metabolism, increasing the pGluAP activity in the soluble fraction of liver, with the consequence of the overproduction of cHP.

Several orexigenic peptides, such as enkephalins (ENK), are able to modify the intake behavior and the energy metabolism in animals [48,49]. A high fat diet changes the hypothalamic expression of this peptide, increases the propensity to overeat, the gain of body weight, and the excess of white adipose tissue [48]. Enkephalin concentration changes in the hepatic tissue and plasma during liver disorders [47], and the liver cells can express Met-ENK immunoreactivity, suggesting that ENK is an endogenous opioid that is produced by the liver [74]. Ours results suggest an increase in soluble tyrosyl aminopeptidase activity (TyrAP), which was implicated in the degradation of ENK in the livers of the Bch group, which could have been related to the differences in food intake and body weight gain in these animals. In fact, ENK was recently proposed as a potential therapeutic peptide for HFD-induced obesity and metabolic disorders [75].

The renin–angiotensin system (RAS) is an important regulator of blood pressure and water balance. Beside the RAS, other local systems have been described in several tissues, including the liver [24,74,76,77]. Changes in the balance of different angiotensin bioactive peptides (AngII, AngIII, AngIV, Ang2-10, and Ang1-7) have been implicated in different chronic diseases, such as liver fibrosis, portal hypertension, hepatic tumors [37,78,79], and metabolic alterations, whereas the blocking of angiotensin receptors has been shown to improve glycemic control and reduce hepatic triglyceride levels [80]. The relative amounts of angiotensines are regulated by the activity of several aminopeptidases, namely angiotensinases (Figure 3A). It is known that these activities are modulated by dietary fat [5,29,30,54,81,82]. Previous results have demonstrated that liver angiotensinases are altered by the thyroid status [83] and obesity [84,85]. The present results confirm the effect of a diet that is enriched with virgin olive oil on liver aminopeptidase activity, increasing the activity of alanyl and cystinyl aminopeptidase. Alanyl aminopeptidase (angiotensinase M) is implicated in the metabolism of AngIII to AngIV [86], whereas cystinyl aminopeptidase (IRAP/AT4) has been described as the insulin regulated aminopeptidase (IRAP) and also as the specific receptor to bind AngIV [83,85,87,88]. This result could indicate an increase of local levels of AngIV, the peptide that binds to the AT4 receptor that is colocalized with the glucose transporter GluT4 [27]. These changes were observed in the membrane-bound fraction, which suggest a more specific functional role for these enzymes [87].

**Figure 6:**
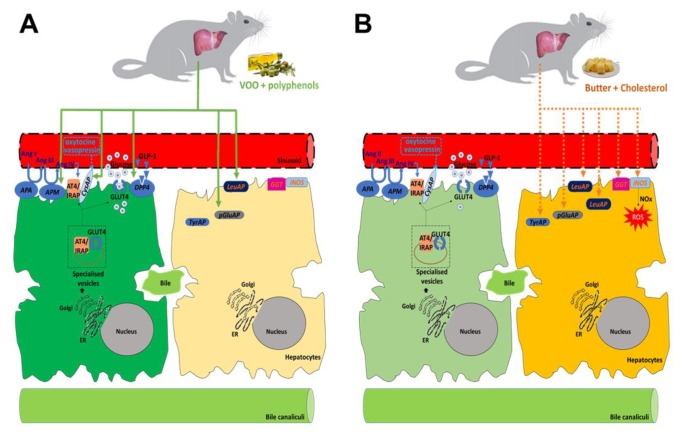
Schematic diagram modeling the role of the high-fat diets (HFDs) that are associated with changes in hepatic peptidases activities. (**A**) The virgin olive oil diet increased the hepatic aminopeptidase activities that are related to glucose metabolism (membrane-bound dipeptidyl peptidase 4 (DPP4), angiotensinase M (Alanine aminopeptidase), insulin-regulated-aminopeptidase (IRAP)/CysAP). (**B**) The butter plus cholesterol diet (Bch) increased soluble and/or membrane-bound activities that are implicated in the control of food intake (tyrosine aminopeptidase (TyrAP)), markers of hepatic damage (leucine aminopeptidase (LeuAP) and gamma glutamyl transferase (GGT)), and the expression of hepatic iNOS, which is involved the reactive oxidative stress (ROS) pathway and induces an increase of plasmatic nitrogen oxides (NOx) levels.

Otherwise, the diet that was enriched with butter and cholesterol increased peptidase activities that have been widely proposed as hepatic biomarkers (GGT and LeuAP) [89,90]. Moreover, the activity levels of the GGT in the liver were significant and positively correlated with plasma triglycerides and total cholesterol. It has been reported that HFDs significantly increased GGT activity, whereas antioxidants (vitamin E) and hypocaloric diets decreased GGT levels [91,92]. Published data have shown that HFDs are the major triggers for oxidative stress [92], and both the cytoprotective role of NO in the liver and hepatic iNOS expression could be an adaptive response to minimize inflammatory injury, hepatic tumor, and cirrhosis [60]. Recently, studies that treated non-alcoholic fatty liver disease (NAFLD) showed that the hepatic expression of iNOS was markedly increased in HFD-fed mice [40,93]. In our results, only animals that were fed with the Bch diet significantly increased the plasmatic levels of nitrate, nitrites, and iNOS expression in the liver, probably as a response to the oxidative stress damage. The main effects of HFDs on peptidase activities are summarized in Figure 6.

## 5. Conclusions

In conclusion, these results demonstrate that the metabolic response to a high fat diet that is enriched with different fat sources could be related to changes in hepatic peptidases that are implicated in the regulation of glucose metabolism and oxidative stress. The diet that was supplemented with butter plus cholesterol altered peptidases activities that are associated with the impaired in the control of food intake and hepatic damage, and it increased body weight. However, the diet that was supplemented with virgin olive oil affected peptidases that are involved in glucose homeostasis (DPP4 and angiotensinases), but it did not alter body weight. Taken together, these results support the beneficial effect of virgin olive oil on energy metabolism and body weight control. Further studies should be performed in order to analyze the expression of key enzymes in fatty acids metabolism, and its relation to peptidase activities.

## Supplementary Materials

The following are available online at https://www.mdpi.com/2072-6643/12/3/636/s1, Table S1: Composition of diets; Table S2: Substrates for Aminopeptidases incubation media; Table S3: Antibodies used for Western-blotting.

## References

[1] Guyenet S.J., Schwartz M.W. (2012). Regulation of Food Intake, Energy Balance, and Body Fat Mass: Implications for the Pathogenesis and Treatment of Obesity. J. Clin. Endocrinol. Metab..

[2] Eckel R.H., Grundy S.M., Zimmet P.Z. (2005). The metabolic syndrome. Lancet.

[3] Pi-Sunyer F.X. (2002). The Obesity Epidemic: Pathophysiology and Consequences of Obesity. Obes. Res..

[4] Hariri N., Thibault L. (2010). High-fat diet-induced obesity in animal models. Nutr. Res. Rev..

[5] Villarejo A., Ramírez-Sánchez M., Segarra A.B., Martínez-Cañamero M., Prieto I. (2015). Influence of Extra Virgin Olive Oil on Blood Pressure and Kidney Angiotensinase Activities in Spontaneously Hypertensive Rats. Planta Medica.

[6] Gao M., Ma Y., Liu D. (2015). High-Fat Diet-Induced Adiposity, Adipose Inflammation, Hepatic Steatosis and Hyperinsulinemia in Outbred CD-1 Mice. PLoS ONE.

[7] Liu X.J., Wang B.W., Zhang C., Xia M.Z., Chen Y.H., Hu C.Q., Wang H., Chen X., Xu D.X. (2015). Vitamin D Deficiency Attenuates High-Fat Diet-Induced Hyperinsulinemia and Hepatic Lipid Accumulation in Male Mice. Endocrinology.

[8] Jiang B., Le L., Wan W., Zhai W., Hu K., Xu L., Xiao P. (2015). The Flower Tea Coreopsis tinctoria Increases Insulin Sensitivity and Regulates Hepatic Metabolism in Rats Fed a High-Fat Diet. Endocrinology.

[9] Carnevale R., Silvestri R., Loffredo L., Novo M., Cammisotto V., Castellani V., Bartimoccia S., Nocella C., Violi F. (2018). Oleuropein, a component of extra virgin olive oil, lowers postprandial glycaemia in healthy subjects. BJCP.

[10] Domínguez-Vías G., Segarra A., Martínez-Cañamero M., Ramírez-Sánchez M., Prieto I. (2017). Influence of a virgin olive oil versus butter plus cholesterol-enriched diet on testicular enzymatic activities in adult male rats. Int. J. Mol. Sci..

[11] Martínez N., Prieto I., Hidalgo M., Segarra A.B., Martínez-Rodríguez A., Cobo A., Ramírez M., Gálvez A., Martínez-Cañamero M. (2019). Refined versus Extra Virgin Olive Oil High-Fat Diet Impact on Intestinal Microbiota of Mice and Its Relation to Different Physiological Variables. Microorganisms.

[12] Nocella C., Cammisotto V., Fianchini L., D’Amico A., Novo M., Castellani V., Stefanini L., Violi F., Carnevale R. (2017). Extra Virgin Olive Oil and Cardiovascular Diseases: Benefits for Human Health. Endocr. Metab. Immune.

[13] Prieto I., Hidalgo M., Segarra A.B., Martínez-Rodríguez A.M., Cobo A., Ramírez M., Abriouel H., Gálvez A., Martínez-Cañamero M. (2018). Influence of a diet enriched with virgin olive oil or butter on mouse gut microbiota and its correlation to physiological and biochemical parameters related to metabolic syndrome. PLoS ONE.

[14] Romani A., Ieri F., Urciuoli S., Noce A., Marrone G., Nediani C., Bernini R. (2019). Health Effects of Phenolic Compounds Found in Extra-Virgin Olive Oil, By-Products, and Leaf of *Olea europaea* L.. Nutrients.

[15] Yubero-Serrano E.M., Lopez-Moreno J., Gomez-Delgado F., Lopez-Miranda J. (2019). Extra virgin olive oil: More than a healthy fat. Eur. J. Clin. Nutr..

[16] Elias S., Wisam S., Luai A., Massad B., Nimer A. (2017). Lipotoxicity in Obesity: Benefit of Olive Oil. Adv. Exp. Med. Biol..

[17] Prieto P., Cancelas J., Villanueva-Peñacarrillo M., Valverde I., Malaisse W. (2005). Effects of an olive oil-enriched diet on plasma GLP-1 concentration and intestinal content, plasma insulin concentration, and glucose tolerance in normal rats. Endocrine.

[18] Nauck M., Homberger E., Siegel E.G., Allen R.C., Eaton R.P., Ebert R., Creutzfeldt W. (1986). Incretin Effects of Increasing Glucose Loads in Man Calculated from Venous Insulin and C-Peptide Responses*. J. Clin. Endocrinol. Metab..

[19] Nauck M., Stöckmann F., Ebert R., Creutzfeldt W. (1986). Reduced incretin effect in type 2 (non-insulin-dependent) diabetes. Diabetologia.

[20] Lovshin J.A., Drucker D.J. (2009). Incretin-based therapies for type 2 diabetes mellitus. Nat. Rev. Endocrinol..

[21] Hare K.J., Vilsbøll T., Asmar M., Deacon C.F., Knop F.K., Holst J.J. (2010). The glucagonostatic and insulinotropic effects of glucagon-like peptide 1 contribute equally to its glucose-lowering action. Diabetes.

[22] Mentlein R., Gallwitz B., Schmidt W.E. (1993). Dipeptidyl-peptidase IV hydrolyses gastric inhibitory polypeptide, glucagon-like peptide-1(7-36)amide, peptide histidine methionine and is responsible for their degradation in human serum. Eur. J. Biochem..

[23] Lugari R., Cas A.D., Ugolotti D., Barilli A.L., Camellini C., Ganzerla G.C., Luciani A., Salerni B., Mitternperger F., Nodari S. (2004). Glucagon-like Peptide 1 (GLP-1) Secretion and Plasma Dipeptidyl Peptidase IV (DPP-IV) Activity in Morbidly Obese Patients Undergoing Biliopancreatic Diversion. Horm. Metab. Res..

[24] Barrett A.J., Rawlings N.D., Woessner J.F. (2004). Handbook of Proteolytic Enzymes.

[25] de Gasparo M., Whitebread S., Bottari S.P., Levens N.R., Pbmwm T., Wexler R.R. (1994). Heterogeneity of Angiotensin Receptor Subtypes. Medicinal Chemistry of the Renin-Angiotensin System.

[26] García-Sáinz J.A., Martínez-Alfaro M., Romero-Avila M.T., González-Espinosa C. (1997). Characterization of the AT1 angiotensin II receptor expressed in guinea pig liver. J. Endocrinol..

[27] Chai S.Y., Fernando R., Peck G., Ye S.Y., Mendelsohn F.A.O., Jenkins T.A., Albiston A.L. (2004). What’s new in the renin-angiotensin system? The angiotensin IV/AT4 receptor. Cell. Mol. Life Sci..

[28] Coleman J.K., Krebs L.T., Hamilton T.A., Ong B., Lawrence K.A., Sardinia M.F., Harding J.W., Wright J.W. (1998). Autoradiographic identification of kidney angiotensin IV binding sites and angiotensin IV-induced renal cortical blood flow changes in rats. Peptides.

[29] Segarra A.B., Ramirez M., Banegas I., Alba F., Vives F., de Gasparo M., Ortega E., Ruiz E., Prieto I. (2008). Dietary Fat Influences Testosterone, Cholesterol, Aminopeptidase A, and Blood Pressure in Male Rats. Horm. Metab. Res..

[30] Segarra A.B., Ruiz-Sanz J.I., Ruiz-Larrea M.B., Ramírez-Sánchez M., de Gasparo M., Banegas I., Martínez-Cañamero M., Vives F., Prieto I. (2011). The Profile of Fatty Acids in Frontal Cortex of Rats Depends on the Type of Fat Used in the Diet and Correlates with Neuropeptidase Activities. Horm. Metab. Res..

[31] de Macêdo S.M., Antunes-Guimarães T., Feltenberger J.D., Santos S.H.S. (2014). The role of renin-angiotensin system modulation on treatment and prevention of liver diseases. Peptides.

[32] Afroze S.H., Munshi M.K., Martínez A.K., Uddin M., Gergely M., Szynkarski C., Guerrier M., Nizamutdinov D., Dostal D., Glaser S. (2015). Activation of the renin-angiotensin system stimulates biliary hyperplasia during cholestasis induced by extrahepatic bile duct ligation. Am. J. Physiol. Gastrointest. Liver Physiol..

[33] Hayden M.R., Sowers K.M., Pulakat L., Joginpally T., Krueger B., Whaley-Connell A., Sowers J.R. (2011). Possible Mechanisms of Local Tissue Renin-Angiotensin System Activation in the Cardiorenal Metabolic Syndrome and Type 2 Diabetes Mellitus. Cardiorenal Med..

[34] Takeshita Y., Takamura T., Ando H., Hamaguchi E., Takazakura A., Matsuzawa-Nagata N., Kaneko S. (2008). Cross talk of tumor necrosis factor-alpha and the renin-angiotensin system in tumor necrosis factor-alpha-induced plasminogen activator inhibitor-1 production from hepatocytes. Eur. J. Pharmacol..

[35] Orlic L., Mikolasevic I., Lukenda V., Anic K., Jelic I., Racki S. (2015). Nonalcoholic fatty liver disease and the renin-angiotensin system blockers in the patients with chronic kidney disease. Wien. Klin. Wochenschr..

[36] Tao X.R., Rong J.B., Lu H.S., Daugherty A., Shi P., Ke C.L., Zhang Z.C., Xu Y.C., Wang J.A. (2019). Angiotensinogen in hepatocytes contributes to Western diet-induced liver steatosis. J. Lipid Res..

[37] Sansoè G., Aragno M., Wong F. (2019). Pathways of hepatic and renal damage through non-classical activation of the renin-angiotensin system in chronic liver disease. Liver Int..

[38] Valko M., Leibfritz D., Moncol J., Cronin M.T., Mazur M., Telser J. (2007). Free radicals and antioxidants in normal physiological functions and human disease. Int. J. Biochem. Cell Biol..

[39] Abbas A., Sakr H.F. (2013). Simvastatin and vitamin E effects on cardiac and hepatic oxidative stress in rats fed on high fat diet. J. Physiol. Biochem..

[40] Pan X., Wang P., Luo J., Wang Z., Song Y., Ye J., Hou X. (2015). Adipogenic changes of hepatocytes in a high-fat diet-induced fatty liver mice model and non-alcoholic fatty liver disease patients. Endocrine.

[41] Taylor B.S., Alarcon L.H., Billiar T.R. (1998). Inducible nitric oxide synthase in the liver: Regulation and function. Biochemistry.

[42] Scharfmann R., Aratan-Spire S. (1991). Ontogeny of two topologically distinct TRH-degrading pyroglutamate aminopeptidase activities in the rat liver. Regul. Pept..

[43] Ra K.S., Suh H.J., Choi J.W. (2012). Hypoglycemic effects of Cyclo (His-Pro) in streptozotocin-induced diabetic rats. Biotechnol. Bioproc. E.

[44] Arechaga G., Prieto I., Segarra A.B., Alba F., Ruiz-Larrea M.B., Ruiz-Sanz J.I., de Gasparo M., Ramirez M. (2002). Dietary fatty acid composition affects aminopeptidase activities in the testes of mice. Int. J. Androl..

[45] Mendez I.A., Ostlund S.B., Maidment N.T., Murphy N.P. (2015). Involvement of Endogenous Enkephalins and β-Endorphin in Feeding and Diet-Induced Obesity. Neuropsychopharmacology.

[46] Cieśla A., Mach T., Pierzchała-Koziec K., Skwara P., Szczepański W. (2006). Met-enkephalin in the liver as a marker of hepatocellular damage in chronic viral hepatitis type B and C. Adv. Med. Sci. Poland.

[47] Owczarek D., Garlicka M., Pierzchała-Koziec K., Skulina D., Szulewski P. (2003). Met-enkephalin plasma concentration and content in liver tissue in patients with primary biliary cirrhosis. Przegl. Lek..

[48] Karatayev O., Gaysinskaya V., Chang G.Q., Leibowitz S.F. (2009). Circulating triglycerides after a high-fat meal: Predictor of increased caloric intake, orexigenic peptide expression, and dietary obesity. Brain. Res..

[49] Hill-Pryor C., Dunbar J.C. (2006). The Effect of High Fat-Induced Obesity on Cardiovascular and Physical Activity and Opioid Responsiveness in Conscious Rats. Clin. Exp. Hypertens..

[50] Fernández D., Valdivia A., Irazusta J., Ochoa C., Casis L. (2002). Peptidase activities in human semen. Peptides.

[51] HOMA2 Calculator: Overview. The Oxford Centre for diabetes endocrinoly and metabolism. Diabetes Trials Unit, HOMA Calculator. (n.d.). https://www.dtu.ox.ac.uk/homacalculator/.

[52] Alba F., Arenas J.C., Lopez M.A. (1995). Properties of rat brain dipeptidyl aminopeptidases in the presence of detergents. Peptides.

[53] Ramírez M., Prieto I., Banegas I., Segarra A.B., Alba F. (2011). Neuropeptidases. Methods Mol. Biol..

[54] Segarra A.B., Arechaga G., Prieto I., Ramirez-Exposito M.J., Martinez-Martos J.M., Ramirez M., Alba F., Ruiz-Larrea M.B., Ruiz-Sanz J.I. (2002). Effects of dietary supplementation with fish oil, lard, or coconut oil on oxytocinase activity in the testis of mice. Arch. Androl..

[55] Bradford M.M. (1976). A rapid and sensitive method for the quantitation of microgram quantities of protein utilizing the principle of protein-dye binding. Anal. Biochem..

[56] Prasad C., Imrhan V., Juma S., Maziarz M., Prasad A., Tiernan C., Vijayagopal P. (2015). Bioactive Plant Metabolites in the Management of Non-Communicable Metabolic Diseases: Looking at Opportunities beyond the Horizon. Metabolites.

[57] Scharfmann R., Ebiou J., Morgat J., Aratan-Spire S. (1990). Thyroid status regulates particulate but not soluble TRH-degrading pyroglutamate aminopeptidase activity in the rat liver. Acta Endocrinol..

[58] Boozer C.N., Schoenbach G., Atkinson R.L. (1995). Dietary fat and adiposity: A dose-response relationship in adult male rats fed isocalorically. Am. J. Physiol. Endocrinol. Metab..

[59] Ghibaudi L., Cook J., Farley C., van Heek M., Hwa J.J. (2002). Fat Intake Affects Adiposity, Comorbidity Factors, and Energy Metabolism of Sprague-Dawley Rats. Obes. Res..

[60] Blundell J.E., Gillett A. (2001). Control of Food Intake in the Obese. Obes. Res..

[61] Iossa S., Lionetti L., Mollica M.P., Crescenzo R., Botta M., Barletta A., Liverini G. (2003). Effect of high-fat feeding on metabolic efficiency and mitochondrial oxidative capacity in adult rats. Br. J. Nutr..

[62] Pérez-Martínez P., García-Ríos A., Delgado-Lista J., Pérez-Jiménez F., López-Miranda J. (2011). Mediterranean diet rich in olive oil and obesity, metabolic syndrome and diabetes mellitus. Curr. Pharm. Des..

[63] Soriguer F., Almaraz M.C., Ruiz-de-Adana M.S., Esteva I., Linares F., García-Almeida J.M., Morcillo S., García-Escobar E., Olveira-Fuster G., Rojo-Martínez G. (2009). Incidence of obesity is lower in persons who consume olive oil. Eur. J. Clin. Nutr..

[64] Polley K.R., Miller M.K., Johnson M., Vaughan R., Paton C.M., Cooper J.A. (2018). Metabolic responses to high-fat diets rich in MUFA v. PUFA. Br. J. Nutr..

[65] Shin S., Ajuwon K.M. (2018). Effects of Diets Differing in Composition of 18-C Fatty Acids on Adipose Tissue Thermogenic Gene Expression in Mice Fed High-Fat Diets. Nutrients.

[66] Oi-Kano Y., Kawada T., Watanabe T., Koyama F., Watanabe K., Senbongi R., Iwai K. (2007). Extra virgin olive oil increases uncoupling protein 1 content in brown adipose tissue and enhances noradrenaline and adrenaline secretions in rats. J. Nutr. Biochem..

[67] Castro-Barquero S., Lamuela-Raventós R.M., Doménech M., Estruch R. (2018). Relationship between Mediterranean Dietary Polyphenol Intake and Obesity. Nutrients.

[68] Pérez-Durillo F., Segarra A.B., Villarejo A., Ramírez-Sánchez M., Prieto I. (2018). Influence of Diet and Gender on Plasma DPP4 Activity and GLP-1 in Patients with Metabolic Syndrome: An Experimental Pilot Study. Molecules.

[69] Cancelas J., Prieto P.G., Villanueva-Peñacarrillo M.L., Valverde I., Malaisse W.J. (2006). Effects of an olive oil-enriched diet on glucagon-like peptide 1 release and intestinal content, plasma insulin concentration, glucose tolerance and pancreatic insulin content in an animal model of type 2 diabetes. Horm. Metab. Res..

[70] Rocca A.S., LaGreca J., Kalitsky J., Brubaker P.L. (2001). Monounsaturated fatty acid diets improve glycemic tolerance through increased secretion of glucagon-like peptide-1. Endocrinology.

[71] Song M.K., Rosenthal M.J., Song A.M., Yang H., Ao Y., Yamaguchi D.T. (2005). Raw vegetable food containing high cyclo (his-pro) improved insulin sensitivity and body weight control. Metabolism.

[72] Hwang I.K., Go V.L.W., Harris D.M., Yip I., Kang K.W., Song M.K. (2003). Effects of cyclo (his-pro) plus zinc on glucose metabolism in genetically diabetic obese mice. Diabetes Obes. Metab..

[73] Araujo R.L., Andrade B.M., Padrón A.S., Gaidhu M.P., Perry R.L.S., Carvalho D.P., Ceddia R.B. (2010). High-Fat Diet Increases Thyrotropin and Oxygen Consumption without Altering Circulating 3,5,3′-Triiodothyronine (T3) and Thyroxine in Rats: The Role of Iodothyronine Deiodinases, Reverse T3 Production, and Whole-Body Fat Oxidation. Endocrinology.

[74] Bergasa N.V., Boyella V.D. (2008). Liver derived endogenous opioids may interfere with the therapeutic effect of interferon in chronic hepatitis C. Med. Hypotheses.

[75] Suo J., Zhao X., Guo X., Zhao X. (2018). Met-enkephalin improves metabolic syndrome in high fat diet challenged mice through promotion of adipose tissue browning. Toxicol. Appl. Pharm..

[76] Fukasawa K.M., Fukasawa K., Kanai M., Fujii S., Harada M. (1996). Molecular cloning and expression of rat liver aminopeptidase B. J. Biol. Chem..

[77] Nagasaka T., Nomura S., Okamura M., Tsujimoto M., Nakazato H., Oiso Y., Nakashima N., Mizutani S. (1997). Immunohistochemical localization of placental leucine aminopeptidase/oxytocinase in normal human placental, fetal and adult tissues. Reprod. Fertil. Dev..

[78] Shim K.Y., Eom Y.W., Kim M.Y., Kang S.H., Baik S.K. (2018). Role of the renin-angiotensin system in hepatic fibrosis and portal hypertension. Korean J. Intern. Med..

[79] van den Hoven A.F., Smits M.L.J., Rosenbaum C.E.N.M., Verkooijen H.M., van den Bosch M.A.A.J., Lam M.G.E.H. (2014). The effect of intra-arterial angiotensin II on the hepatic tumor to non-tumor blood flow ratio for radioembolization: A systematic review. PLoS ONE.

[80] Graus-Nunes F., de Santos F.O., de Marinho T.S., Miranda C.S., Barbosa-da-Silva S., Souza-Mello V. (2019). Beneficial effects of losartan or telmisartan on the local hepatic renin-angiotensin system to counter obesity in an experimental model. World J. Hepatol..

[81] Min L., Sim M.K., Xu X.G. (2000). Effects of des-aspartate-angiotensin I on angiotensin II-induced incorporation of phenylalanine and thymidine in cultured rat cardiomyocytes and aortic smooth muscle cells. Regul. Pept..

[82] Reaux A., Fournie-Zaluski M.C., David C., Zini S., Roques B.P., Corvol P., Llorens-Cortes C. (1999). Aminopeptidase A inhibitors as potential central antihypertensive agents. Proc. Natl. Acad. Sci. USA.

[83] Segarra A.B., Prieto I., Martínez-Cañamero M., de Gasparo M., Luna J.d.D., Ramírez-Sánchez M. (2018). Thyroid Disorders Change the Pattern of Response of Angiotensinase Activities in the Hypothalamus-Pituitary-Adrenal Axis of Male Rats. Front. Endocrinol..

[84] Gajdosechova L., Krskova K., Segarra A.B., Spolcova A., Suski M., Olszanecki R., Zorad S. (2014). Hypooxytocinaemia in obese Zucker rats relates to oxytocin degradation in liver and adipose tissue. J. Endocrinol..

[85] Prieto I., Segarra A.B., de Gasparo M., Martínez-Cañamero M., Ramírez-Sánchez M. (2018). Divergent profile between hypothalamic and plasmatic aminopeptidase activities in WKY and SHR. Influence of beta-adrenergic blockade. Life Sci..

[86] Villarejo A.B., Segarra A.B., Ramírez M., Banegas I., Wangensteen R., de Gasparo M., Cobo J., Alba F., Vives F., Prieto I. (2012). Angiotensinase and vasopressinase activities in hypothalamus, plasma, and kidney after inhibition of angiotensin-converting enzyme: Basis for a new working hypothesis. Horm. Metab. Res..

[87] Prieto I., Villarejo A.B., Segarra A.B., Wangensteen R., Banegas I., de Gasparo M., Vanderheyden P., Zorad S., Vives F., Ramírez-Sánchez M. (2015). Tissue distribution of CysAP activity and its relationship to blood pressure and water balance. Life Sci.

[88] Segarra A.B., Prieto I., Martinez-Canamero M., Vargas F., De Gasparo M., Vanderheyden P., Zorad S., Ramirez-Sanchez M. (2018). Cystinyl and pyroglutamyl-beta-naphthylamide hydrolyzing activities are modified coordinately between hypothalamus, liver and plasma depending on the thyroid status of adult male rats. J. Physiol. Pharmacol..

[89] Kanno T., Maekawa M., Kanda S., Kohno H., Sudo K. (1984). Evaluation of Cytosolic Aminopeptidase in Human Sera: Evaluation in Hepatic Disorders. Am. J. Clin. Pathol..

[90] Porta M., Pumarega J., Guarner L., Malats N., Solà R., Real F.X., PANKRAS II Study Group (2012). Relationships of hepatic and pancreatic biomarkers with the cholestatic syndrome and tumor stage in pancreatic cancer. Biomarkers.

[91] Bezerra-Duarte S.M., Faintuch J., Stefano J.T., Sobral de Oliveira M.B., de Campos Mazo D.F., Rabelo F., Vanni D., Nogueira M.A., Carrilho F.J., Marques Souza de Oliveira C.P. (2014). Hypocaloric high-protein diet improves clinical and biochemical markers in patients with nonalcoholic fatty liver disease (NAFLD). Nutr. Hosp..

[92] Li Q., Feenstra M., Pfaffendorf M., Eijsman L., van Zwieten P.A. (1997). Comparative Vasoconstrictor Effects of Angiotensin II, III, and IV in Human Isolated Saphenous Vein. J. Cardiovasc. Pharmacol..

[93] Hassanin A., Malek H.A., Saleh D. (2014). Heparin modulation on hepatic nitric oxide synthase in experimental steatohepatitis. Exp. Ther. Med..

