# Supplementary material for: The Role of High Fat Diets and Liver Peptidase Activity in the Development of Obesity and Insulin Resistance in Wistar Rats
Source: Nutrients. 2020 Feb 28;12(3):636. doi: 10.3390/nu12030636 (PMC7146256; doi:10.3390/nu12030636)
Supplement: Supplementary file 1 [file nutrients-12-00636-s001.pdf]

Table 1: Composition of diets (supplementary material)

	S			VOO			Bch		
Components	g/100g	kJ/100g	% Total E	g/100g	kJ/100g	% Total E	g/100g	kJ/100g	% Total E
NFE	60	1003.2	72.1	48	802.6	43.4	48	802.6	43.9
Fiber	4			3			3		
Protein	16.5	275.9	19.8	13	217.4	11.8	14	234.1	12.8
Minerals (Ash)	5			5			5		
Lipids	3	112.9	8.1	22	827.6	44.8	21	790	43.3
SFA	0.75	28.22	2.0	3.74	140.7	7.6	13.02	489.8	26.8
MUFA	0.50	18.81	1.4	17.38	653.8	35.4	7.56	284.4	15.5
PUFA	1.75	65.84	4.7	0.88	33.1	1.8	0.42	15.8	0.9
Cholesterol							1.50		
Total E		1392			1848			1827	

Table 2: Substrates for Aminopeptidases incubation media (supplementary material)

INCUBATION SOLUTION (100 ml)								
Enzyme	Substrates (µM)	BSA (mM)	DTT (mM)	EDTA (µM)	$MnCl_2$ (mM)	$CaCl_2$ (mM)	Buffer	
							Phosphate (50 mM)	Tris-HCl (50 mM)
AlaAP	Ala-β-NA 100	1.5	0.65				pH 7.4	
ArgAP	Arg-β-NA 100	1.5	0.65				pH 7.4	
AspAP	Asp-β-NA 100	1.5				$3 \cdot 10^{-8}$		pH 7.4
DPP4	H-Gly-Pro-4-Methoxy-β-NA 100	1.5	0.65				pH 8.3	
GluAP	α-Glu-β-NA 100	1.5	0.65		50			pH 7.4
GGT	γ-Glu-β-NA 100	1.5	0.65				pH 7.4	
IRAP	Cys-β-NA 100	1.5	0.65					pH 6.0
LeuAP	Leu-β-NA 100	1.5	0.65				pH 7.4	
pGluAP	pGlu-β-NA 100	1.5	0.65	10.1			pH 7.4	
TyrAP	Tyr-β-NA 100	1.5	0.65				pH 7.4	

Table 3: Antibodies used for Western-Blotting (supplementary material)

**Peptide/protein target**	**Antigen sequence (if known)**	**Name of Antibody**	**Manufacturer, catalog #, and/or name of individual providing the antibody**	**Species raised in; monoclonal or polyclonal**	**Dilution used**
iNOS		polyclonal Rabbit Anti-iNOS/NOS Type II	BD Transduction Laboratories, 610333	rabbit; polyclonal	1:500
α-tubulin		anti- alpha tubulin	Invitrogen Cat# 32-2500	mouse, monoclonal	1:5,000
anti-rabbit		peroxidase-conjugated rabbit antibody	Vector Labs	goat; polyclonal	1:2,000
mouse IgG		horseradish peroxidase (HRP)-conjugated goat anti-mouse IgG (H+L)	Jackson ImmunoResearch Laboratories, PA, USA. Catalog # 115-035-003	goat; polyclonal	1:1,000
